# Administration of Strategic Agreements in Public Hospitals: Considerations to Enhance the Quality and Sustainability of Mergers and Acquisitions

**DOI:** 10.3390/ijerph18084051

**Published:** 2021-04-12

**Authors:** Javier Cerezo-Espinosa de los Monteros, Antonio Castro-Torres, Juan Gómez-Salgado, Javier Fagundo-Rivera, Carlos Gómez-Salgado, Valle Coronado-Vázquez

**Affiliations:** 1 Andalusian Ministry of Health and Families, 41020 Seville, Spain; juanj.cerezo.sspa@juntadeandalucia.es; 2 Andalusian Health Service, 41071 Seville, Spain; antonio.castro.torres.sspa@juntadeandalucia.es; 3 Department of Sociology, Social Work and Public Health, Faculty of Labour Sciences, University of Huelva, 21007 Huelva, Spain; 4 Safety and Health Postgraduate Program, Universidad Espíritu Santo, Guayaquil 091650, Ecuador; 5 Health Sciences Doctorate School, University of Huelva, 21007 Huelva, Spain; javier.fagundo308@alu.uhu.es; 6 Centro Universitario de Enfermería Cruz Roja, University of Seville, 41009 Seville, Spain; 7 Faculty of Labour Sciences, University of Huelva, 21007 Huelva, Spain; cargomsal@gmail.com; 8 Illescas Health Centre, Castilla-La Mancha Health Service, 45200 Toledo, Spain; mvcoronado@msn.com; 9 Health Science Institute of Aragon, 50009 Zaragoza, Spain; 10 Department of Health Sciences, Santa Teresa de Jesus Catholic University of Avila, 05005 Avila, Spain

**Keywords:** mergers and acquisitions, sustainability, cooperation, healthcare systems, health management, innovation, staff health, quality of care, culture, strategy

## Abstract

Merger processes between hospitals have high benefit potential for patients, staff and managers. This integration of health centres can improve the quality and safety in patient care. Additionally, cooperative processes enhance the sustainability of the health system, by increasing team spirit, giving innovative ideas and improving staff satisfaction. In this article, the critical factors for successful hospital mergers and acquisitions in the Public Health System were considered to develop a brief guide to help with the organisation of a merger process. Five sections were designed: Strategic administration and objectives, Staff management, New hospital complex structure, Processes and Results. This guide facilitates the communication between a variety of stakeholders, thus improving the engagement between all members of the new healthcare system. This could be particularly important for countries with large regional variance in the organisation of health care and resources.

## 1. Introduction

### 1.1. General Contextualisation of the Topic

Many reasons lead us to study the effectiveness of hospital mergers. They have become very frequent in recent years, although failures are still very common. Hospital mergers have many potential benefits, such as economies of scale, greater purchasing power, more possibilities for the acquisition of expensive equipment, opportunities to share high-cost technology, the creation of hyperspecialised units for specific pathologies, and more [1]. They can also impair patients’ perception of quality and satisfaction [2]. However, mergers and acquisitions can also raise a climate of distrust between managers and clinical professionals, with poorer workers’ satisfaction and higher rates of absenteeism [3]. Market concentration can also be a problem as, in many cases, the expected high efficiency based on economies of scales is not always achieved [4]. All of these factors have made the success of hospital mergers an appealing subject of interest. With this evidence, many aspects have been considered to have such an influence on the success of mergers, but more research is still necessary. Furthermore, the high complexity of the subject, the many dimensions to take into account, the different sources of uncertainty, and the existence of conflicting interests between different stakeholders make appropriate the use of a multi-criteria decision-analysis approach [5].

### 1.2. Background

Hospital integrations have been very frequent since the 1990s, both in the United States and in Europe. Indeed, more than half of the hospitals in the United States and in Europe have become a member of a partnership, system or network with other hospitals [6]. Specifically, several countries had a great increase in mergers in the last years. In the United States, after the Affordable Care Act implementation [7,8], the integration of the health systems has grown quickly, comprising 81% of acute care hospitals, in parallel to mergers among insurers, pharmacies, and pharmaceutical companies [9]. In England, 112 out of 223 acute hospitals merged between 1997 and 2006 [10]. In Sweden, since the nineties, the number of hospitals has been halved [11]. In France, over 90 mergers between private hospitals have happened since 1995 [12]. As economic crisis was one important driver of mergers, it can be expected that this trend towards increasing the number of mergers will go on, something that makes finding out significant factors for its success even more relevant [13].

By now, however, mergers still show very high rates of failure. Studies conclude that, in Europe and North America, most mergers fail because of different reasons such as differences in corporate cultures [14,15], or not appropriately planning the structure and processes of the merged institution [16,17]. Therefore, it seems that the merger processes happened very fast, before having thoroughly planned the significant milestones [18]. Additionally, many research projects have tried separately to identify potential factors to improve a merging system.

Keane, Sutton, Farragher and Long [19] conducted a review about mergers of hospitals in several countries. They found that a key factor to foster hospital mergers was putting patients’ needs first [20,21], rather than focusing publicly on economic potential benefits. Moreover, as a merger process is a period of uncertainty for most workers, emphasizing the priority of providing high quality care for patients can enhance the adoption of innovative practices and reinforce the autonomy of professionals, giving them a sense of control [19]. Other factors that fostered mergers were the involvement of staff in decision making and collaborative conversations between senior management boards and hospital staff. Avoiding uncertainty that could threaten professional identity was also an important factor as clinicians focused on improving patient’s health [22,23].

Besides, hospital mergers can make available a higher number of physicians for the same clinical process, and more possibilities to choose for patients. Thus, patients could have more accessibility to hyperspecialised professionals in a concrete clinical process, yet in different places. In the USA, for example, the possibility to choose a physician has been rated in the satisfaction surveys within a range from “somewhat” to “very” important [24]. Although better patient experience may not always correlate with higher clinical quality, patient perception of quality increases when consumers can select their healthcare providers. Therefore, ensuring these aspects could be considered critical for merger success [25].

On the other way, despite the potential benefits of consolidations, such as increased efficiency and lower costs, researchers and regulators have raised concerns about the increased market concentration effect, as well as the potential for higher prices that stem from lower levels of competition. This could be a key factor to make the merger become an antitrust case [26,27]. As an evidence for this, Lewis and Pflum [28], based on acquisitions occurring across the U.S. during 2000–2010, found that the negotiated prices of hospitals acquired by out-of-market systems increased by about 17% more than at independent hospitals. In Germany, hospital mergers were subject to antitrust law and should be prohibited if the merged entity obtains more than 40% of the market share or if the merger leads to a significant level of concentration [29]. Dafny, Ho and Lee [30], using data on USA hospital mergers from 1996–2012, found that within-state hospital mergers yielded price increases of 7–9% for acquiring hospitals, whereas out-of-state acquisitions did not yield significant increases. Gaynor, Laudicella and Propper [31] found that consolidation reduces potential competition, which has been shown in the UK market to have some beneficial effects on patient outcomes and length of stay. They conclude that the English government should carefully consider potential losses before allowing more mergers between short term general hospitals. Market concentration may even have a negative impact on the patient’s perception of quality, as mentioned above. If patients, during their hospital stay or post-discharge, have not received the necessary resources (because the hospital was not able to provide them or they were out-of-market), it can be inferred that with fewer competitors and more market concentration there is less incentive to keep patients’ satisfaction [24]. Nevertheless, recent investigations on the merger of rural hospitals have been associated with increased capital expenditure after merging [32].

As staff satisfaction was positively correlated with health care quality, higher job satisfaction and a rise in overall quality are key factors to persuade staff about the benefits of a merger [15]. However, Gaynor et al. [31] pointed out that the increasing effect of a hospital merger on staff satisfaction was reduced and ephemeral, which might help to explain the standstill of healthcare quality after a merger. A possible factor related to this effect was the huge challenge of the necessary organizational change after a merger, sometimes associated with adverse staff health and increased sickness absence [33]. This has even been associated with an increase in the prescription of psychotropic medication and antidepressants [34]. Other studies found that immediately after a merger announcement, the employees’ number of absence days due to sickness increased to definite or long-term absence. Higher global stress has also been reported, and significant uncertainty about situations such as job loss, leadership change or placement change [35,36,37,38]. In this way, healthcare workers need to know the challenges and support available to prepare for a merger and be able to adapt to the new working context. Sensitive personal factors such as the active participation of professionals must be considered. Therefore, it seems that including proper management of human factors in merger planning may be critical [39].

### 1.3. Aim of the Study

Besides this, the implementation in two merged hospitals of common work procedures, both in the clinical and non-clinical areas, seems to be an important tool to achieve a successful hospital restructuring [7]. Discovering the appropriate ways to elaborate and implement these common procedures can help to plan the merger.

Therefore, after our previous considerations, the next step should be to investigate the factors necessary to build a successful cooperation between hospitals, increasing their effectiveness and raising workers’ self-esteem, to ensure that potential quality and economic benefits of the mergers will become real. This research seems particularly important when we consider that hospitals require the largest proportion of health care expenditure in almost all the Organization for Economic Co-operation and Development countries [40].

Given this evidence, our article aims to provide a practical and brief guide for planning a merger between hospitals of the public administration, considering key factors for a successful integration. This guide can be very useful in improving the process of planning a merger, and for personnel in this planning process from the outset, ensuring that critical success factors were considered from the first step.

## 2. Methodology

### 2.1. Study Design

Firstly, a preliminary search was carried out for literature on hospital merger protocols and case reports of relevant hospital mergers, fusions, and acquisitions, which highlighted the main factors that led to their success, difficulties, or failure of their integration. To do this, inclusion and exclusion criteria were established, and data were extracted from several sources in an organised manner, including healthcare databases, scientific journals, scientific societies’ websites, and healthcare organisations’ websites. Additionally, manual searches in the reference lists of the selected documents were completed. Evidence was found to be scarce, partial, and sometimes contradictory, with insufficient research on the subject. Therefore, to complement the lack of evidence, it was decided to combine three methodologies in this work: (1) reviewing the evidence on merger processes; (2) examining and extracting success factors from the evidence; and (3) consulting experts to confirm or modify these factors.

As mentioned, a literature review was conducted to find relevant key factors in order to elaborate a compilation of factors for the consideration of the experts. The opinions of the experts were gathered through an online survey (see Section 2.3). Finally, after having gathered the evidence and the experts’ points of views, the guide was elaborated synthesising literature evidence with experts’ opinions. The guide is compounded by items representing each of the success factors identified and short explanations of the rationale below each item.

### 2.2. Literature Review

#### 2.2.1. Preliminary Search

The preliminary literature review showed that merger processes, in general, include several collaborative scenarios, with different levels of cooperation, and all of them have been taken into account in this work, having been categorised as follows: (1) concrete collaborations: agreements in some areas, as well as joint purchase from providers, common use of equipment or buildings, shared education programmes, or shared clinical protocols; (2) strategic alliances: collaborations have expanded to most of the areas of the hospitals, although both centres continue being autonomous, with two different boards and budgets; (3) complete mergers: an organisation completely integrated, with one board and budget, common human resources, shared objectives, and most of the hospital departments merged [41].

Merger is a dynamic process over time that needs to consider a number of relevant dimensions [34]. Among the main categories established for the inclusion criteria, our main pillars were strategic vision, governance, leadership, values and culture of the organisation, organisation of the economic aspects of hospitals, integration of clinical services, joining information systems, and merging support functions. Additionally, political pressure for or against the merger, willingness of professionals to cooperate, economic resources, physicians’ and patients’ opinions, and management skills were identified as important factors.

#### 2.2.2. Selected Studies

A total of 1630 studies were found in the database search, and 13 of these papers were finally selected after checking that they met the inclusion criteria and referred to relevant hospital merger processes: 1 article from Germany, 7 from the USA, 1 from Spain, 2 from the United Kingdom, and 2 from Sweden (Table 1).

#### 2.2.3. Identified Success Factors

Following this review of the literature, the factors for a successful integration identified in each study are shown in Table 2. Additionally, the factors that were mentioned more than once were identified in Table 3. These factors were provided to the experts to collect their opinions (as can be seen in Section 2.3).

### 2.3. Gathering Experts’ Opinions

To complement the information that was found after the literature search, opinions were collected from experts who had experienced hospital integration processes first-hand. Criteria for selecting experts were defined, this being a profile of a top hospital manager (for example, a CEO or a medical director), who in the previous years had responsibilities over planning, implementing, and leading a merger between two hospitals at the Spanish health care system. Using the technique of snowball sampling, a first expert who had been a colleague of one of the investigators was approached and was asked to answer the survey and to suggest new possible experts. This way, current and former CEOs of the University Hospital Complex of Granada, the University Hospital Complex of Huelva, the Hospital Complex of Navarra, and the Virgen Macarena-Virgen del Rocio Hospitals of Seville, all in Andalusia (Spain), were invited to participate in this process. These four managers had been involved in past merger processes, from the preliminary phases and planning, during the implementation phase, to the final result of the merger.

In order to use a semi-structured interview technique, a questionnaire was designed using Google Forms (Google, Mountain View, CA, USA), and sent to every expert through email, asking for their extensive opinion about several questions. Simultaneously, the success factors found in the literature search were provided to the experts, to inform their judgement. The questions were chosen based on the literature review findings and the experience of the researchers, as they had previously taken part in 2 mergers. This questionnaire consisted of the questions stated in Table 4.

Subsequently, the experts’ responses were summarised so that most frequently repeated opinions could be taken into account for the final guide. An opinion was considered frequently repeated when at least 3 of the 4 experts had proposed it. This way, it was confirmed that success factors identified in the literature were useful to elaborate the guide. Additionally, the information provided by the experts was used to explain deeply and in detail the rationale of every item considered in the guide.

## 3. Results

The success factors for hospital mergers identified, after synthesising information gathered from literature review and experts’ opinions, are the following:

### 3.1. Establishing a New Management Structure That Reflects the Merged Institution

Top leadership should be strongly involved, with enough power, avoiding competition between the leaders during the merger. Integration of governance and leadership and establishing a new management structure that takes into account the different merged centres are key. The new structure must represent the merging strategy (e.g., there is a position with responsibilities over managing merger difficulties). If a university is involved, it is important that it is represented too, so that the two medical schools could work towards common ends. The lines of work have to be sufficiently institutionalised to persist through the changes in organisational leadership that will eventually occur. The new power structure has to be established quickly [42,44,45,46,48,49,50,51].

### 3.2. Developing a Strategy for Managing Differences in Culture and Values

Having a strategy for the integration of culture and values is important, avoiding competition between both hospitals. Taking into account the differences in leadership styles, work culture, patterns of communication, and delegating tasks is key, so that both hospitals aspire towards a near-identical ideal of the future preferred culture. It is necessary to avoid the competition between managerialism and professionalism, and the alignment of academic and clinical cultures [46,47,50,51,52,54].

### 3.3. Involving Healthcare Workers and Intermediate Managers in Decisions That Affect Them

Management at all levels is important for the development and outcome of the merger process. It seems to provide better results in an incremental, emergent, bottom-up approach. It is important to obtain professional buy-in and to deal with the resistance from the professionals, horizontal tensions, clashes and other hindering factors, avoiding threats for salaries, positions and titles [43,44,47,49,52,54]. The merger must be seen as a complex interplay of internal and external factors as well as actors. The main stakeholders from the population have to be involved. Achieving common objectives with local authorities can facilitate the merger. The political opposition’s efforts to further their own agendas in government can be a key factor [43,44,50,51,54].

### 3.4. A Communication Process with Relevant and Timely Information for Healthcare Workers through Different Channels

Strong feelings in workers should be managed: hope, fear, frustration, resentment, concerns… It is especially important to provide quality and accurate information to manage rumours quickly [43,44,47,50].

### 3.5. Developing a Common Clinical Strategy for the Merged Hospital

The integration of strategic vision is important to bring about a common clinical strategy for the merged hospital. Adopting an evolutionary rather than revolutionary approach is helpful. Additionally, planning the milestones for the utilisation of complementary clinical capabilities with a clear definition of roles for each hospital and fostering cordial relations is important, while at the same time safeguarding and promoting clinical specialisation [44,46,47,50].

### 3.6. Establishing an Information System Appropriate for the Merged Institution

Integration of information systems is particularly important, and having shared information for decisions, e.g., a balanced scorecard system mapping out quality, financial, and other strategic indicators for both hospitals. The tools and interventions developed must be available to all hospitals. This also makes evaluating the merger process possible, learning from the implementation, and correcting deviations [42,46,47,49].

### 3.7. Improving Quality with Higher Volumes of Patients or Rationalisation of Processes

Standardising processes in the large volume areas and when appropriate allows improvement in the quality of care. The integration of some clinical services facilitates the establishment of high-volume programs procedures, which consistently produce better patient outcomes. It is also possible to take advantage of being a bigger centre to attract new appealing projects. The perception of improvements from the merger is essential to maintain inter-organisational and workers’ stability [42,45,51,53].

### 3.8. Integrating Competing Units

The integration of clinical services is challenging and important. Establishing collaboration lines with departments in the other hospital for more easy working is important. Sharing devices and equipment can be attractive. Additionally, different types of fusion can be considered before the complete merger of hospitals [42,46,47,51].

### 3.9. Taking Advantage of Merged Education Programs

The merger can result in common programs with a depth and quality that will make it among the best in its class, improving training and development by working together, and making the merger more attractive for professionals [45,47,48,51].

### 3.10. Putting Emphasis on the Benefits of the Integration

In the communication process, it is important to allow the hospital to realise many of the benefits of clinical integration [44,51,53].

### 3.11. Aligning Allocation of Resources with the Merger Success

The integration of financial systems and support functions is important. It is possible to better finance those service lines that succeeded in developing a single, integrated strategic business plan for both centres, and reach administrative cost savings. Reaching significant economies of scale, shared purchasing, and using common several services and departments can be a facilitating factor. It is also helpful to use performance-based salaries to enhance the merger [44,45].

### 3.12. Becoming a Reference Hospital for More Patients

Merging the capabilities of both hospitals can lead to more prestigious services among the population and healthcare professionals, becoming a reference hospital for more patients [42,45].

### 3.13. Geographic Proximity for Merged Hospitals

Geographic proximity between the merged hospitals is a facilitating factor, as it is easier to establish relationships between professionals from both hospitals and working at both hospitals at the same time. This facilitates the emergence of a new common professional identity [45,47].

### 3.14. Reaching Increased Efficiency

It is important to set realistic expectations about potential synergies and growth, with realistic objectives in terms of savings in management costs by taking into account the amount of managerial input needed to implement the merger, and not underestimating the amount of time needed for restructuring [42,45].

### 3.15. Sharing Good Practices across Constituent Trusts

A key factor is the adoption of best practices, which brings recognition to workers and facilitates mergers [51,52].

## 4. The Guide

The final product of this study was a brief guide to plan the merger of two hospitals. This guide must be completed by hospital clinical leaders, leaders of non-clinical areas, and top management teams of the integrated centres. In this way, the process of planning the merger is participatory from the beginning, and every relevant stakeholder is considered (see Supplementary File).

The guide consists of five sections: (1) Strategic direction and objectives, (2) People management, (3) New integrated hospital structure, (4) Processes, (5) Results.

### 4.1. Details of the Guide

Below, the parameters that define each section of the guide are described. For each section, all of the relevant items of a concrete merger must be completed. When all items have been completed, a roadmap for merging the centres will be available. The different roadmaps elaborated by each stakeholder will be sent to the top management team, and they will integrate the different contributions into a final global roadmap. In this guide, the items that must be completed are accompanied by the rationale behind them, so that reflections are easier. These items are the following:

a. Establishing the strategic direction and objectives to be achieved during the merger planning (1) Explaining which concrete centres shall be joined, (2) Describing the rationale behind why centres must be joined, (3) Deciding what degree of union will be achieved in both centres after the merger is completed for the different areas of the hospitals, (4) Illustrating the organisational horizon the hospitals are moving towards, (5) Specifying objectives to reach on the following dimensions (Patients, Professionals, The organisation, scheduling the objectives).

b. Deciding how human resources will be managed (1) What will be done to decrease competition between groups and increase mutual esteem, (2) How to facilitate the creation of a new common identity for professionals from both hospitals, (3) Establishing a communication plan, specifying information to be provided to every relevant audience, and its timing; (4) Considering the cultural differences between both centres, and which specific actions should be done to get them closer.

c. Defining the structure of the new joined centre after the integration (1) Composition of the management team, (2) Structure of staff positions and departments leadership, (3) Explaining how the objectives to be reached by the whole hospital, by each hospital department and by each clinician and non-clinical worker should be aligned with the merger structure and strategy.

d. Defining which clinical and non-clinical processes should be unified.

e. Explaining what results are intended to be obtained after the integration is complete (1) Regarding management and care indicators, (2) Regarding the external image of the new organisation.

### 4.2. Rationale of the Experts behind the Items

The following is a brief explanation of the rationale of items. In the guide, they have been explained more deeply.

First, the strategic direction and objectives to be achieved are established. To do this, which centres merge, why they do so, what degree of merging they will have, and what is the organisational horizon (the vision) must be explained. As the experts said:

*…designing new objectives (general, individual, of healthcare, scientific), that can only be reached by the new structure…*

The objectives should also be specified to patients, professionals, and the organisation, as well as the time horizon during which the necessary work to merge both centres will be done. The key factor to consider in this first section is “the corporate culture” existing in the centres and the sense of belonging of professionals. Interviewed experts thought that:

*(patients gained) …equity, access to the same services no matter the town where the patients live…*

*…the phase of cooperation should last 12-18 months, the phase of strategic alliance about 2 years, and the merger phase also 12-18 months…*

Therefore, we would be talking about hospital areas, since this sense of belonging will be more commonly applied to the whole area rather than to the particular centre where these professionals work. Although each hospital maintains its identity in some way, a higher identity appears to encompass both centres.

*…sharing some objectives and resources in common, but without losing the identity of each centre, developing shared units and services…*

If the main impetus for integration stems from political authority, it is essential to incorporate these authorities into the most relevant decision-making processes. This plays a key role in bringing a clear horizon closer to citizens, explaining what the objectives and benefits of joint work are. Experts said that it was important:

*…from the beginning, making clear the support of political authorities…*

The management and intermediate office structures are joined, common work processes are created, and joint projects are established to achieve objectives focused on patients, quality of care, accessibility, and improved resource management such as beds, technology, etc. A greater volume of activity in some pathologies would allow greater specialisation of professionals, thus improving the quality of care. Additionally, there may be greater stability in attendance, as there would be more professionals prepared to attend the same process. Experts thought it was important:

*…explaining to population a clear horizon, without uncertainties, explaining the objectives of improvement and the costs…*

Secondly, it is necessary to explain how people will be managed, defining what will be done to decrease competition between groups and increase mutual esteem, thus helping to bring up a new common identity. Some topics that deserve special attention are the workload and the existence of wage differentials (working hours, night shifts...), as well as differences in employment management (authorised time reductions, percentage of eventual and permanent contracts). For these cases, it is appropriate to homogenise the organisational criteria.

*…differences in culture don´t need to be changed, but instead it is better to bring closer both hospitals and getting used and comfortable with the differences…*

It is important for professionals to have frequent participation in the different work commissions and forums. A “bottom-up” approach is recommended, where many of the most important integration projects have been drawn by professionals. It is essential to collect the opinion of all stakeholders, as well as explain the advantages of integration to all the involved professionals. To provide and exchange information, it is important to keep social networks, as well as the different media and information, for professionals and the general population.

*…doctors and nurses are the most important groups and should be involved in committees, many forums, with a wide and numerous representation…citizens and patients through the healthcare workers, using the participation forums of clinical units…*

Thirdly, how the structure of the new centre will look after integration must be explained, defining the composition of the management team and the structure of intermediate positions. It is also necessary to record how the objectives of hospitals, care units, and professionals will be aligned with the converged structure and strategy. To generate the new culture of the “new large hospital complex”, it is necessary to promote mutual learning, rotations, stays in the other hospital, create mixed units, and work to align the organisational horizon behind integration, organisational structure, goals, and corporate communication. Aligning all these factors is one of the fundamental elements for the success of the merger.

*…management structure, rules, reorganizing clinical areas, new profile for the centres, changing the portfolio, common objectives for clinical units, unifying the electronic health record, standardization of information systems, a unified external image…*

Fourth, it is necessary to define which processes in the clinical and non-clinical fields should be unified. Clinical and economic processes, administrative and general service processes can be shared. Unifying work processes involves joint workgroups.

Finally, what results are intended to be achieved after the merger process is complete must be clearly defined, both regarding management and assistance indicators as well as the external image of the new organisation.

*…it is better, and it causes less problems beginning the merger by other areas different from clinical departments, for example industrial processes (equipment, patient transfers, joint protocol, etc.).*

How will we know that the merger of the two centres has been successful? Showing it through specific indicators. This section requires the development of a dashboard to know whether the merger process is progressing on the intended path, and which partial and final evaluations of the process will be carried out. To do this, it is advisable to integrate, or at least make compatible, the information systems and Information technologies of both centres.

*(having available) …aggregate data, for an intermediate process, that is complex, but can provide the necessary information for monitoring the process….*

## 5. Discussion

As mentioned in the introduction, the key policies for quality patient care during a merger process are respect for patient choice policies, conservation and improvement of hospital assets regarding quality and materials, organisational policies for costs and investment, and selective and personalised attention to chronic patients [12]. Our research shows the main considerations about an optimised hospital merger that coincide with the considerations of the World Health Organization [55]: (1) The costs, patient’s outcomes and satisfaction depend, among others, on the country and market; (2) Bigger hospitals are not necessarily better, the optimal hospital size depends on local healthcare needs, distance to resources, and the availability of complementary services; (3) Accurate objectives and investments need to be measured based on the achievement of better clinical results, and commonly established size indicators.

Providing more accessibility for patients and setting quality as a goal should be critical to contribute to cost control. A cost reduction can be achieved if the process leads to a common organisational culture and a more thorough control of the provided services. Cost savings could help in the development of the hospital itself: i.e., for making major investments in hospital functions or making new buildings [56].

In France, Norway and Portugal, mergers between public hospitals are treated as administrative acts and internal reorganisations of public services. In this case, in France, there have been consequent attempts to combine competition and cooperation between coordinated groups of public hospitals [12]. Thus, hospital competition on quality of care and resource control in these countries mainly depends on being part of a public or private healthcare system because of these proposals for cooperation that exist in the public system.

Other types of strategic mergers are followed to cover isolated areas in rural areas. Normally, before the merger, these patients have to be referred to larger hospitals, therefore increasing the risk of lower quality care of these rural areas [57]. Further, some substantial investments in quality improvement or research may not be allowed. In these cases, mergers and joint collaborations allow participants to combine clinical and/or management strengths and gain greater geographic coverage [58].

To enhance the team culture, moving away from the hierarchical structure towards a team-based culture is necessary, as professionals would need to feel engaged and supported. It is a contrast to this management model to adopt the university-type entrepreneurial culture, based on individual achievements and governance structures [52]. In times of crisis or change, if there is a strong, shared vision of improving patient care with a deliberate focus on engaging all team members, this process can drive towards overall significantly positive results [59]. For this, it is necessary to explore whether the new conditions of the hospital merger can help to attract and retain professionals.

Every organisational change at work seems to have an impact on employees’ health and well-being. The adverse effects are primarily driven by job uncertainty among employees. In these cases, excessive psychotropic prescription rates are found after a change in management or employee lay-off, as such changes may elevate uncertainty about future employment [34]. Hospital mergers have, also, a significant effect on long-term sickness absence of the employees in an early phase of the process and, later, from 2 to 4 years after mergers [33,60,61]. Evidence supports an association between psychosocial stress and risk of depression, anxiety, and disturbed sleep at the beginning of the merger process. A higher rate of early retirement is also experienced after a change of management, merging or relocation of work units [62]. In addition, mergers were also associated with an excess rate of psychotropic prescriptions in the second half of the 12-month follow-up period, which could be hypothetically explained by subsequent reduction in redundant staff following mergers [24].

High nursing turnover has been associated with mergers. Turnover among healthcare personnel may be predominantly negative, and it has been related with deterioration in nurses’ mental health, lower job satisfaction, and lower patient satisfaction with the received care. Higher incomes may indeed be a desired result of a merger, but when services are relocated between merging hospitals, professionals specialising in those services are expected to continue offering the same service. For some, of course, this means moving to another hospital, but the turnover destination matters, and getting off the hospital area represents a loss for the sector [60].

However, the negative effect may not be significant after a year [61]; the workers seem to adapt very quickly to their new situation and, as staff job satisfaction is positively correlated with health care quality, both variables may be ensured if the staff expectations regarding the merger benefits are met during the early years after the integration [15,33,61]. A recent study demonstrated that unit-level upsizing is related with reduced risk of employee sickness absence [63]. Additionally, yet small, the increase in job satisfaction immediately after merger approval indicates the success of pre-merger staff engagement. Therefore, such efforts should be continued and possibly intensified throughout the merger process to prevent the decline in job satisfaction that could compromise healthcare quality.

Sickness absence increases during mergers, but employees return to their job when they have adapted to the ‘shock’ [60]. This also highlights the importance of managers to control the staff expectations so as to minimise any possible post-merger shock [15,19]. It is possible that hospital managers underestimate the difficulties in implementing the change process, leading to overstatement of benefits and unrealistic expectations that are later unmet [15].

### 5.1. Limitations

Regardless of the importance of economic competition for merger success, this issue was not considered in the guide because it was devised for the Spanish public health care system, where competition between hospitals is not a relevant factor, but it should be taken into account when considering mergers in other systems and cost-saving or cost-effectiveness studies play a more important role.

### 5.2. Impact Statement for Policy Makers and Managers

This guide has offered as a theoretical contribution that can help in the development of cooperative public health systems. Key factors for the merger of hospitals have been identified. Therefore, this research can add evidence to similar works in other contexts, trying to find out what factors remain important in different healthcare systems. Future research can start studies based on the factors identified here. Besides, as stated above, despite having focused this research on the Spanish healthcare system, the identified success factors may be useful in other countries with a similar organisation and financing model of health system. Additionally, working with an evidence-based approach, along with experts’ opinions can provide high value when planning new management interventions [64,65]. In addition, using a multi-criteria decision analysis method proves to be useful for decisions so complex as those related to a merger process, and especially for its long-term sustainability [66].

Other practical contributions of this guide are: (1) It allows a participatory process, as professionals may be asked to partake; (2) It allows elaborating a roadmap of the hospitals’ merger process based on relevant evidence; (3) It avoids overlooking key factors for success in mergers; (4) It allows planning the merger process in the long term; (5) It facilitates follow-up and monitoring of the merger process throughout time; (6) It allows carrying out partial and final assessment of the merger process; (7) It facilitates the communication of the merger process protocol to the different stakeholders.

Future research could lead to the improvement of this guide by using multi-criteria decision analysis to identify key factors related to the level of satisfaction of healthcare workers and environmental factors, as well as improving the weights of experts from a more objective perspective [67,68]. Therefore, this guide would be useful to reduce the ambiguity of different labour networks, as well as to look for an approach to the private sector, and even to the integration of primary care facilities and hospitals.

## 6. Conclusions

This manuscript has introduced the report of a guide to help hospital mergers. The guide itself is composed of five sections (strategic direction and objectives, people management, structure of the new hospital complex, processes, and results), encompassing a total of 19 questions that reflect the successful factors for an effective healthcare integration. This research seeks the delimitation of a set of organisational management mechanisms that could strengthen the capacities of the Public System in achieving cost-efficiency through the integration of result-oriented management and the creation of public value.

## Figures and Tables

**Table 1:** Selected studies [42,43,44,45,46,47,48,49,50,51,52,53,54].

Country	Investigation Title and Reference
Germany	Leadership in Health Care: Developing a Post-Merger Strategy for Europe’s Largest University Hospital [42]
USA	Effective Communications: Critical factors in Health Alliance Success [43]
	Model for a merger: New York-Presbyterian’s use of service lines to bring two academic medical centres together [44]
	Camelot or common sense? The logic behind the UCSF/Stanford merger [45]
	Boston University Researchers Formulate Framework for Successful Hospital Mergers and Systems Integration [46]
	Integration of an Academic Medical Centre and Community Hospital: The Brigham and Women’s/Faulkner Hospital Experience [47]
	Failure of the merger of the Mount Sinai and New York University Hospitals and Medical Schools: Part 1 [48]
	A Decade of Preventing Harm [49]
Spain	Determinants of the ex-Post Performance of Mergers and Acquisitions: A Case Study [50]
United Kingdom	Process and impact of mergers of NHS trusts: Multicentre case study and management cost analysis [51]
	Organizational culture and post-merger integration in an academic health centre: A mixed-methods study [52]
Sweden	Is it better to be big? The reconfiguration of 21st century hospitals: Responses to a hospital merger in Sweden [53]
	Competing logics in hospital mergers: The case of the Karolinska University Hospital [54]

**Table 2:** Facilitators of a successful hospital merger [42,43,44,45,46,47,48,49,50,51,52,53,54].

Country	Document	Facilitators of a Successful Hospital Merger
Germany	Leadership in health care: Developing a post-merger strategy for Europe’s largest university hospital [42]	Establishing a new management structure that reflects the merged institution. Preserving the range of services for the entire entity. Integrating competing units into one whole. Managing resistance from health care professionals. Reaching increased efficiency. Becoming a reference hospital for more patients. Taking advantage of new possibilities for research. Improving quality with higher volumes of patients or rationalisation of processes. Establishing an information system appropriate for the merged institution. Aligning performance-based salary with the success of the merger.
USA	Effective Communications: Critical factors in Health Alliance Success [43]	A communication process with relevant and timely information for healthcare workers through different channels. Involving main stakeholders in decisions that affect them. Involving healthcare workers and intermediate managers in decisions that affect them.
	Model for a merger: New York-Presbyterian’s use of service lines to bring two academic medical centres together [44]	Establishing a new management structure that reflects the merged institution. Developing a common clinical strategy for the merged hospital. A communication process with relevant and timely information for healthcare workers through different channels. Putting emphasis on the benefits of the integration. Involving healthcare workers and intermediate managers in decisions that affect them. Involving main stakeholders in decisions that affect them. Aligning allocation of resources with the merger success. Adopting an evolutionary rather than revolutionary approach.
	Camelot or common sense? The logic behind the UCSF/Stanford merger [45]	Establishing a new management structure that reflects the merged institution. Becoming a reference hospital for more patients. Geographic proximity for merged hospitals. Reaching increased efficiency. Improving quality with higher volumes of patients or rationalisation of processes. Providing more options for patients through the merger. Aligning allocation of resources with the merger success. Taking advantage of merged education programs.
	Boston University Researchers Formulate Framework for Successful Hospital Mergers and Systems Integration [46]	Establishing a new management structure that reflects the merged institution. Developing a common clinical strategy for the merged hospital. Developing a strategy for managing differences in culture and values. Integrating competing units into one whole. Establishing an information system appropriate for the merged institution. Integrating financial systems. Integrating support functions.
	Integration of an Academic Medical Centre and Community Hospital: The Brigham and Women’s/Faulkner Hospital Experience [47]	Taking advantage of merged education programs. Developing a common clinical strategy for the merged hospital. Geographic proximity for merged hospitals. A clear definition of roles for each hospital. Involving healthcare workers and intermediate managers in decisions that affect them. A communication process with relevant and timely information for healthcare workers through different channels. Developing a strategy for managing differences in culture and values. Integrating competing units into one whole. Planning the milestones. Establishing an information system appropriate for the merged institution.
	Failure of the merger of the Mount Sinai and New York University Hospitals and Medical Schools: Part 1 [48]	Establishing a new management structure that reflects the merged institution. Taking advantage of new possibilities for research. Taking advantage of merged education programs. Avoiding threats for salaries, positions, and titles.
	A Decade of Preventing Harm [49]	Establishing an information system appropriate for the merged institution. Involving healthcare workers and intermediate managers in decisions that affect them.
Spain	Determinants of the ex-Post Performance of Mergers and Acquisitions: A Case Study [50]	Establishing a new management structure that reflects the merged institution. Developing a common clinical strategy for the merged hospital. Developing a strategy for managing differences in culture and values. Involving main stakeholders in decisions that affect them. A communication process with relevant and timely information for healthcare workers through different channels.
United Kingdom	Process and impact of mergers of NHS trusts: Multicentre case study and management cost analysis [51]	Establishing a new management structure that reflects the merged institution. Developing a strategy for managing differences in culture and values. Putting emphasis on the benefits of the integration. Improving quality with higher volumes of patients or rationalisation of processes. Sharing good practices across constituent trusts. Involving main stakeholders in decisions that affect them. Integrating competing units into one whole. Taking advantage of merged education programs. Avoiding unnecessary delays in decisions for the merger, and not underestimating the amount of time needed for restructuring. Setting realistic objectives on efficiency improvement.
	Organizational culture and post-merger integration in an academic health centre: A mixed-methods study [52]	Developing a strategy for managing differences in culture and values. Sharing good practices across constituent trusts. Involving healthcare workers and intermediate managers in decisions that affect them.
Sweden	Is it better to be big? The reconfiguration of 21st century hospitals: Responses to a hospital merger in Sweden [53]	Putting emphasis on the benefits of the integration. Considering different types of integration intensity. Improving quality with higher volumes of patients or rationalisation of processes.
	Competing logics in hospital mergers: The case of the Karolinska University Hospital [54]	Involving main stakeholders in decisions that affect them. Involving healthcare workers. Developing a strategy for managing differences in culture and values.

**Table 3:** Number of articles citing facilitating factors.

Facilitating Factor	Number of Articles Citing This Factor
Establishing a new management structure that reflects the merged institution.	8
Developing a strategy for managing differences in culture and values.	6
Involving healthcare workers and intermediate managers in decisions that affect them.	6
A communication process with relevant and timely information for healthcare workers through different channels	4
Developing a common clinical strategy for the merged hospital.	4
Establishing an information system appropriate for the merged institution.	4
Improving quality with higher volumes of patients or rationalization of processes.	4
Integrating competing units into one whole.	4
Taking advantage of merged education programs.	4
Putting emphasis on the benefits of the integration.	3
Aligning allocation of resources with the merger success.	2
Becoming a reference hospital for more patients.	2
Geographic proximity for merged hospitals.	2
Reaching increased efficiency.	2
Sharing good practices across constituent trusts.	2

**Table 4:** Interview questions.

Group of Experts’ Questions Script
A healthcare organization can be formed by various hospitals. The number of hospitals to be merged can affect the complexity of the process. Some authors recommend not merging all hospitals at the same time but working at the beginning just with 2 of them. What do you think about this recommendation?
The drive for merging the hospitals could come from political authorities. Can you make some suggestions about how political authorities can facilitate the process?
What suggestions can you make about geographical distance of the merging hospitals?
The integration of 2 hospitals can happen with various degrees of union, from concrete collaborations to a complete merger. Besides, there can be different degrees of union for the departments of the hospitals. What can you suggest about the degree of union so that this is successful?
What suggestions can you make to get the merger to provide an attractive organizational horizon for healthcare professionals?
What suggestions can you make to get the merger to provide an attractive organizational horizon for the community?
How could the merger increase the prestige of research capabilities?
Probably, the merger will make possible concentrate in the same clinical unit a higher number of patients with the same disease. This allows clinical professionals to improve their experience. How could this help to the merger success?
When covering staff positions at the hospitals, as the merger made the centre bigger, it could be easier to attract talent. How could this be used to facilitate the merger process?
Some authors recommend aligning management tools with the merger process success. Regarding this, what suggestions can you make?
Why is the merger better for the patients instead of maintaining the same previous structure?
Why is the merger better for healthcare workers instead of maintaining the same previous structure?
Why is the merger better for healthcare organizations instead of maintaining the same previous structure?
A merger usually lasts several years, and during that time, several milestones need to be considered. Which do you think are the main milestones and the different phases?
How long do you think it necessary to consider the merger process completed?
What can be done to decrease prejudices and negative stereotypes of professionals of one hospital as regards the other hospital’s professionals?
What can be done to avoid competitive behaviours between merged hospitals?
What are specific ways to promote professionals’ involvement in the merger process? Please, provide some examples.
About relations regarding unions, what´s your advice for facilitating the merger?
How could communication processes facilitate the merger (communication plan, contents, target population, time, etc.)?
One of the most difficult challenges for mergers is cultural differences (leadership styles, delegating tasks, degree of professionals’ involvement, communication style)? What suggestions can you make to avoid these difficulties?
It is probable that the managing team structure will change after the merger. What can you recommend so that the new structure facilitates the merger process?
Intermediate manager positions can also be merged. How do you recommend doing this so that the integration process is facilitated?
Elaborating common procedures for clinical and non-clinical processes can be very helpful. What do you suggest so that this can facilitate the merger?
It could be useful to have a balance scorecard with indicators that allows evaluate the merger process. What’s your advice for this?
What could be done to promote a joint external image of the new merged organization for the general population?
Finally, is there something important we didn´t ask for and you would like to comment on?

## Data Availability

All data is available within this article and its Supplementary Material.

## Supplementary Materials

The following are available online at https://www.mdpi.com/article/10.3390/ijerph18084051/s1, Guide to Planning Hospital Mergers.
