# Supplementary material for: Administration of Strategic Agreements in Public Hospitals: Considerations to Enhance the Quality and Sustainability of Mergers and Acquisitions
Source: Int J Environ Res Public Health. 2021 Apr 12;18(8):4051. doi: 10.3390/ijerph18084051 (PMC8069692; doi:10.3390/ijerph18084051)
Supplement: Supplementary file 1 [file ijerph-18-04051-s001.pdf]

GUIDE TO PLANNING HOSPITAL MERGERS

This instrument serves to assess the key aspects that are to be considered during the merger design as well as during the merger process.

It is organised by sections according to specific objectives. Firstly, the strategic direction and objectives to be achieved is established. To do this, which centres are integrated, why they are being integrated, what degree of union they will have, and what is the organisational horizon they move towards is stated. Also, the objectives about patients, professionals and the organisation are specified, as well as the time horizon to work on the integration of both centres.

Secondly, how people will be managed, what will be done to decrease competition between groups and to increase mutual esteem is explained. This will help to bring up a new common identity and set up a communication plan considering cultural differences between both centres.

Thirdly, the structure of the new centre after the integration will be defined, stating the composition of the management team, as well as the structure of intermediate positions. Also, how the objectives of hospitals, care units and professionals will be aligned with the merger structure and strategy will be explained.

Fourthly, which clinical and non-clinical processes are convenient to be unified will be defined.

Finally, the intended results to be achieved after the merger process is complete will be described.

A. SETTING THE STRATEGIC DIRECTION AND THE OBJECTIVES TO BE ACHIEVED

A.1.- SCOPE

Mark the hospital centres that will establish some degree of cooperation

- Cooperation strategies will be established between 2 centres: Yes No
- Cooperation strategies will be established between more than 2 centres: Yes No

If yes, indicate the number:

- If you will be working with more than 2 centres, please say in which 2 centres you will start:

A.1.1.- Background of the merger of both hospitals.

- The main driver for integration originates in political authority: Yes No
- Actions will be taken to incorporate these authorities into making the most relevant decisions: Yes No
- All stakeholders that have influenced the integration will be taken into account: Yes No
- When deciding on integration, the previous existence of common projects between both hospitals has been taken into account: Yes No
- When deciding on integration, economic aspects have been taken into account: Yes No
- When deciding on integration, geographical aspects have been taken into account: Yes No
- Existing means of transport for both professionals and the population to easily move between both centres are sufficient: Yes No
- A common medical history will be established: Yes No
- Integrated information systems will be established: Yes No
- A comprehensive and integrative logistics plan will be established: Yes No

A.1.2.- Degree of the merger.

- State the phase in which the merger process you are working on is:
  - Occasional collaborations.
  - Strategic alliance (agreements extend to almost all areas of the hospital).
  - Full integration (a new centre is created from the previous two. aparece una identidad superior que engloba a ambos centros.).
- With a lower degree of merger to a full merger, it is possible to achieve many of the pursued objectives (occasional collaborations): Yes No
- Integration needs to be more intensive in specific areas of work: Yes No
- There are 'historical collaborations' that can be useful as a starting point for the merger process: Yes No

A.1.3.- Type of hospital intended to be achieved.

- Indicate all the management tools the hospitals have which can be used to promote collaborative work between them.
- It is possible to increase research prestige due to the sum of the capacities of the centres: Yes No
- More data will be exploited when conducting analysis and research: Yes No
- The merger process can accelerate the translation of research results into the healthcare practice: Yes No
- Attempts will be made to attract a greater number of clinical trials: Yes No
- Research projects will be conducted on less frequent pathologies after grouping these cases from both hospitals: Yes No
- Reference assistance units shall be formed at the regional, national or even international level, with professionals from both centres: Yes No
- Joint work between both centres will be sought to improve indicators of quality, patient safety, productivity, decreased delays, health outcomes, etc. If yes, state which ones:
- By joining the resources of both hospitals, it will be easier for professionals to access:
  - High technology:
  - Advanced therapeutic diagnostic techniques:
  - Specific knowledge areas:
  - High levels of training:
- More Health Sciences students will be able to access training in both centres: Yes No
- Some types of students that were not previously trained in these hospitals will be able to access training in both centres: Yes No

A.2.- OBJECTIVES

A.2.1.- Strategic objectives.

- The activity of specific pathologies will be grouped to achieve a higher quality of care: Yes No
- By assisting a higher number of patients in a single unit, the centre's service portfolio will be expanded: Yes No
- The provision of services with two similar care units will be reordered in both hospitals: Yes No
- Patients with the same type of health care will be treated at the same hospital regardless of their place of residence (improving equity): Yes No
- Patient displacements will be minimised by processes offered at the other hospital: Yes No
- Attempts will be made to reduce the reiteration of tests in both hospitals: Yes No

- Attempts will be made to reduce delays in consultations, to have a diagnostic test, or to undergo a surgery: Yes No
- There will be a greater number of professionals trained to attend the same process: Yes No
- In one of the centres, there is an excess of capacity in some areas, which will be used by the other hospital: Yes No
- Patients waiting for emergency rooms in one hospital will be able to be admitted to the other: Yes No
- A “common beds” occupation system will be established to be used by both hospitals: Yes No
- The integration of both hospitals will be used to offer greater career development opportunities: Yes No
- The integration of both hospitals will be used to help improve the selection of specialists in training: Yes No
- Some hospital head offices will be renewed by taking advantage of natural staff departures (e.g. retirements): Yes No
- The merger of both centres will be used to improve the ability of attracting and retaining professionals: Yes No
- The merger of residents' training programmes will be used to enhance their training: Yes No
- The merger of the training programmes of both centres will be used to enhance undergraduate training: Yes No
- The merger of the training programmes of both centres will be used to enhance postgraduate training: Yes No
- There will be more funding for joint research studies between both centres: Yes No
- Electromedical technology belonging to one of the hospitals may be used by both: Yes No
- Joint agreements with suppliers will be established: Yes No
- The same facilities may be used in joint action: Yes No
- Partners will be unified with Primary Care: Yes No

A.3.- TEMPORARY HORIZON

Specify the most important objectives to be achieved in the different consecutive phases of the process:

- Collaborative phase (12-18 months):
- Strategic alliance phase (2 years):
- Unification phase (12-18 months):

B. PEOPLE MANAGEMENT

- Merger work will begin with the most integration-friendly clinical groups: Yes No
- Working groups will be established with professionals from both hospitals, from which joint work proposals will emerge: Yes No
- Initially, work will be conducted based on objectives with a high probability of success, with the aim of increasing the professionals’ motivation: Yes No
- Success will be celebrated in the merger process: Yes No
- Particular attention will be paid to groups that may be more uncomfortable with the merger process, as they may be more affected by the change: Yes No
- Multiple commissions and joint working forums will be established with healthcare professionals from both centres to encourage their wide and frequent participation: Yes No
- The Medical Boards will participate in the design of the merger project: Yes No
- The Nursing Boards will participate in the design of the merger project: Yes No
- The Staff Boards will participate in the design of the merger project: Yes No

- An advisory commission to the Management Area will be set up for the merger process, made up of opinion leaders from both hospitals: Yes No
- Public debates will be held with the participation of assistance units that have made positive progress in the merger process: Yes No
- Suggestions and views of patients on the merger project will be collected through various mechanisms: Yes No
- Communication channels with professional colleagues will be established: Yes No
- Communication channels with universities will be established: Yes No
- The participation of the media will be facilitated: Yes No
- A technical commission will be set up to support merger in non-care processes (catering/hotel industry, industrial processes, technologies, etc.): Yes No
- The specific areas of trade union intervention will be determined from the outset, as regards changes in working conditions: Yes No
- Current jobs will be guaranteed: Yes No
- A plan to renew and improve resources and working facilities will be promoted, taking advantage of the margins resulting from the merger process: Yes No
- An attempt will be made with the trade unions not to modify the representative capacity of the merged centres in relation to the number of trade union delegates available: Yes No
- Knowledge on the opinion of all stakeholders will be sought during the merger process: Yes No

B.1.- SETTING UP A COMMON SUPRA-IDENTITY

- Working groups will be formed with members from both hospitals in the clinical field: Yes No
- Working groups will be formed with members from both hospitals in the non-clinical field: Yes No
- Working groups will be formed with members from both hospitals at the level of each care unit: Yes No
- Workload differences between both centres will be analysed: Yes No
- The difference in available resources between both centres will be analysed: Yes No
- The existence of pay gaps (on-call shifts, afternoon hours, etc.) between both centres will be analysed: Yes No
- Attempts will be made to homogenise differences in labour management (off-call, authorised half-time working hours, percentage of temporary, interim and fixed contracts, consultations for the same care process with a greater number of patients, usually overburdened on-call units compared to less burdened ones, etc.): Yes No
- Common clinical objectives will be established to benefit both centres at the same time: Yes No
- Common non-clinical objectives will be established to benefit both centres at the same time: Yes No
- Common objectives will be established at the level of each unit, that will benefit both centres at the same time: Yes No
- Both centres will go as one to accreditation processes: Yes No
- Both centres will go as one to research calls: Yes No
- In order to facilitate work between professionals in both centres, video or audio technologies will be used: Yes No
- A new name will be established for the merger of both centres with which both hospitals will be identified: Yes No
- A new logo will be created: Yes No
- A slogan for integration will be created: Yes No

B.2.- COMMUNICATION PLAN

- Face-to-face meetings will be held between managing boards and professionals to report on the merger process: Yes No
- Professionals will have channels to send their suggestions, concerns and opinions to the management board (e.g. a space on the intranet, a specific e-mail address, etc.): Yes No
- The time horizon for the objectives to be achieved at each stage of the merger process will be communicated to the different stakeholders: Yes No
- The integration schedule will be communicated to the different stakeholders at the beginning: Yes No
- The evolution of the planned activities will be communicated to the different stakeholders at each stage: Yes No
- A hotline will be established for any doubts arising concerning the merger, including rumours about the process that may contribute to workers' dissatisfaction or insecurity towards the merger and its implications: Yes No
- One of the members of the management team will be designated as responsible for coordinating the merger process: Yes No
- A participatory commission will be set up to monitor joint work between both hospitals: Yes No
- An initial meeting will be held with all the responsible medical and nursing staff to address the strategy for merger of both hospitals: Yes No
- A space will be created on the hospital intranet to inform about the merger process: Yes No
- A space will be created on the hospital website: Yes No
- In larger municipalities, meetings will be organised to report on the project's main outlines: Yes No
- The project will be communicated to citizens through associative movements: Yes No
- Meetings will be held with the provincial leaders of the most representative parties to inform them of the project: Yes No
- The project will be communicated through the media: Yes No
- A public consultation will be held on a document that summarises the merger project in the patient's language: Yes No
- Where there are groups of patients who may be affected by the changes, specific communication tools will be designed: Yes No

B.3.- WORKING CULTURE

- There are symptoms of the "merger syndrome" (loss of corporate identity, high levels of stress, frequent crises, power struggles, and loss of motivation and commitment): Yes No
- Shifts and working hours in the other hospital will be promoted: Yes No
- The creation of mixed units will be promoted: Yes No
- For each care unit, a steering committee will be created, consisting of professionals from both centres: Yes No

C. STRUCTURE OF THE NEW HOSPITAL COMPLEX

C.1.- STRUCTURE OF THE HOSPITAL HEAD OFFICES

- Define the final structure of the shared management team after the integration:
- Define the final shared structure of intermediate positions after the integration:

C.2.- OBJECTIVES AND STRUCTURE ALIGNMENT

- Some objectives that help merger will be included among the hospitals' general objectives: Yes No
- Some objectives that help merger will be included among the assistance units' objectives: Yes No
- Some objectives that help merger will be included among the non-assistance units' objectives: Yes No

D. PROCESSES

- The merger process will first begin by integrating the administrative services: Yes No
- The user support service will be unified: Yes No
- Dialogue with external suppliers will be unified: Yes No
- The healthcare circuits will be unified: Yes No
- Joint working groups will be created for the unification of work processes: Yes No
- The merger process will begin by unifying the critical and most prevalent processes: Yes No
- Good practices of one hospital will be adopted in the other: Yes No
- That assistance work with a greater volume of activity will be protocolised: Yes No
- The functioning of medical and surgical services working around the same pathology will be integrated: Yes No

E. RESULTS

- Regular reviews of the quantity and quality of care production will be organised in both centres: Yes No
- A management board will be developed to know whether the merger process is moving forward along the intended path, through which partial and final evaluations can be carried out: Yes No
